# Potential Geographic Distribution of the Rare and Endangered Plant *Sauvagesia rhodoleuca* in China Under Climate Change Scenarios

**DOI:** 10.1002/ece3.73295

**Published:** 2026-05-05

**Authors:** Jinxin Wei, Bangze Li, Rong Zou, Weiping Wu, Fei Tan, Li Ding, Tao Ding

**Affiliations:** ^1^ Guangxi Key Laboratory of Plant Conservation and Restoration Ecology in Karst Terrain, Guangxi Institute of Botany, Guangxi Zhuang Autonomous Region and Chinese Academy of Sciences Guilin Guangxi Zhuang Autonomous Region China; ^2^ State Key Laboratory of Plant Diversity and Specialty Crops, Institute of Botany, Chinese Academy of Sciences Beijing China; ^3^ China National Botanical Garden Beijing China; ^4^ University of Chinese Academy of Sciences Beijing China; ^5^ Guangxi Institute of Botany, Guangxi Zhuangzu Autonomous Region and the Chinese Academy of Sciences Guilin Guangxi Zhuang Autonomous Region China; ^6^ Jiuwanshan National Nature Reserve Administration of Guangxi Liuzhou Guangxi Zhuang Autonomous Region China; ^7^ Guilin Xing'an Lijiangyuan Forest Ecosystem Observation and Research Station of Guangxi Guilin Guangxi Zhuang Autonomous Region China

**Keywords:** climate change, environmental factor, MaxEnt model, potential geographic distribution, *Sauvagesia rhodoleuca*

## Abstract

*Sauvagesia rhodoleuca*, a monospecific endemic to China, belongs to the Ochnaceae. It holds significant academic value in studying the evolution and phylogeny of this family's flora, and its rhizomes have medicinal properties. Due to human activities and habitat destruction, the wild population of this species has sharply declined, and it has been listed as a national second‐class protected plant. It is now only sporadically distributed in mid‐low mountain forests of Guangxi and Guangdong at an altitude of 400–1000 m. To reveal its potential distribution and response to climate change, this study optimized parameters of the MaxEnt model using 29 distribution points and 38 environmental variables. The ENMeval package was used to simulate suitable area distributions under current and future (2050s, 2070s) SSP126, SSP245, and SSP585 scenarios. The results showed that the optimized MaxEnt model had AUC values > 0.988, indicating high prediction accuracy. Bio14, T_BS, T_CLAY, and Bio8 were identified as the main environmental drivers, with hydrothermal conditions dominating the distribution pattern. Under current climate conditions, the total suitable habitat area was estimated at 146.81 × 10^4^ km^2^, concentrated in southern China. Projections under future scenarios (2050s and 2070s) across SSP126, SSP245, and SSP585 scenarios indicated significant range expansion, with the most pronounced increase (63.81% expansion) observed under the high‐emission SSP585 scenario in the 2070s. The distribution centroid showed a consistent northward shift, with new suitable habitats emerging primarily in Fujian, Hunan, Jiangxi, and Zhejiang. This study provides a scientific basis for wild resource conservation and cultivation introduction of *S. rhodoleuca*, recommending prioritized conservation of high‐suitability areas like Dayao Mountain National Nature Reserve of Guangxi and cultivation introduction planning in new areas like Hunan and Jiangxi, while considering environmental requirements for medicinal component accumulation.

## Graphical abstract

This study used an optimized MaxEnt model to project the distribution of the endangered Chinese endemic *Sauvagesia rhodoleuca*. Under future climate scenarios, suitable habitat is expected to expand significantly and shift northward, with hydrothermal conditions as key drivers. We recommend prioritizing conservation in Guangxi's Dayao Mountain and planning cultivation introductions in Hunan and Jiangxi, considering both habitat suitability and conditions for medicinal component accumulation.

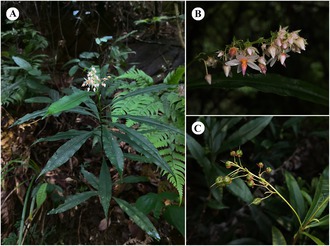

## 1 Introduction

The formation of geographic distribution patterns of plant species is shaped by the combined effects of their biological traits and environmental factors (Thuiller et al. 2005; Gan et al. 2024). Species distribution is influenced by multiple factors, including biological factors, environmental factors, their ability of adaptation and dispersal (Elith and Leathwick 2009), among these factors, climatic factors play a decisive role at global or regional scales (Bohan et al. 2022). They also indirectly affect the spatial distribution characteristics of species by regulating plant phenological and physiological metabolic processes (Marchioro and Krechemer 2021; Dong et al. 2024). Climate change has emerged as one of the most severe threats to global biodiversity. According to the Sixth Assessment Report (AR6) of the Intergovernmental Panel on Climate Change (IPCC), the global surface temperature increased by 1.09°C during 2011–2020 relative to the 1850–1900 baseline period. Projections suggest that by the end of the 21st century, the global mean surface temperature will increase by 1.3°C–2.4°C under the SSP126 scenario and by 3.3°C–5.7°C under the SSP585 scenario (*IPCC* 2023). Concurrent with rising temperatures, annual precipitation is exhibiting an increasing trend, which alters soil moisture and habitat conditions, thereby affecting species growth and population establishment (Bellard et al. 2012; Thackeray et al. 2022; Hosseini et al. 2024). At regional scales, the increased frequency of extreme climatic events under climate change and intensified human activities has already caused significant geographic distribution shifts in numerous species (Ceballos et al. 2015; Pörtner et al. 2022). Approximately half of the assessed species have shown migration trends toward polar or higher altitude regions (Michelozzi and De' Donato 2021; Kikstra et al. 2022), resulting in profound impacts on plant distribution patterns (Coumou and Rahmstorf 2012; Aguirre‐Liguori et al. 2019). Climate change is leading to suitable habitat contraction or shifts for endangered plant species, thereby threatening their sustainable utilization (Jiang et al. 2023; Gan et al. 2024). Consequently, elucidating the species‐environment relationship and predicting potential species distributions are of critical importance for biodiversity conservation.

Species distribution models (SDMs) are the most widely used tools for identifying potential species distributions and environmentally suitable habitats, with extensive applications in biodiversity conservation (Elith and Leathwick 2009; Booth 2016). Among the various SDM methods, such as bioclimatic modeling (BIOCLIM), domain environmental envelope (Domain), climate change experiment (CLIMEX), genetic algorithm for rule‐set production (GARP), ecological niche factor analysis (ENFA), and maximum entropy (MaxEnt). MaxEnt has been recognized as the most effective approach due to its high predictive accuracy and low sample size requirements (Elith et al. 2006; Phillips et al. 2006; Xiang et al. 2025). Particularly for rare and endangered species with only point‐based occurrence data (such as herbarium or museum records), MaxEnt can effectively handle small sample sizes and achieve accurate predictions for species with localized distributions and narrow environmental tolerance ranges (Elith et al. 2011). This model not only visually demonstrates the adaptive thresholds of key environmental factors for species through response curves but also effectively balances model complexity and goodness of fit using regularization parameters (Elith and Leathwick 2009; Elith et al. 2011; Zhang et al. 2025). Although default parameter settings may lead to overfitting or model redundancy (Warren et al. 2014), posing particular challenges for modeling endangered species with narrow habitats and scarce data (Thuiller et al. 2005; Chichorro et al. 2019), parameter optimization can significantly enhance its predictive performance (Kass et al. 2021). Therefore, MaxEnt has been widely applied to assess the impact of climate change on species distribution, particularly for habitat evaluation and planning in endangered species conservation and biosphere reserves (Guevara et al. 2017; Khadka and James 2017; Jiang et al. 2020; Li et al. 2023).

*Sauvagesia rhodoleuca* (Diels) M. C. E. Amaral, belongs to the *Sauvagesia* Linnaeus (Ochnaceae), a monospecific genus of evergreen shrubs endemic to China (Zhang and Amaral 2007). *Sauvagesia rhodoleuca* exhibits high morphological similarity to the South American genus *Wallacea*, thereby holding significant academic value for studying the evolution, biogeography, and phylogeny of Ochnaceae (Miao et al. 2020). The roots and stems of *S. rhodoleuca* possess medicinal properties, exhibiting efficacy in itch relief and insecticidal activity. However, due to deforestation, habitat fragmentation, and overharvesting, wild populations have experienced dramatic declines (Miao et al. 2020). Consequently, it has been listed as a National Class II Protected Wild Plant (Miao et al. 2020; Chen et al. 2023). This species is restricted to scattered populations in lower to mid‐montane forests or forest margins within Guangxi and Guangdong Provinces, occurring at elevations of 400–1000 m. It preferentially grows on sandy soils near valley streams, exhibiting a narrow distributional range (Miao et al. 2020). Research on *S. rhodoleuca* has primarily focused on its taxonomy and morphological characteristics (Amaral 2006; Zhang and Amaral 2007), habitat conditions (Chen et al. 2023), reproductive biology (Chai et al. 2010; Zeng et al. 2010; Chen et al. 2023), and community characteristics (Chen et al. 2016). Nevertheless, studies predicting its potential distribution under climate change scenarios are still lacking, which is crucial for developing scientifically sound conservation strategies for this endangered endemic plant.

Based on specimen records, field survey data, and multi‐scenario environmental variables, this study optimized the MaxEnt model parameters using the ENMeval R package. By integrating ArcGIS, we simulated the potential distribution of *S. rhodoleuca* habitats under current and future climate scenarios, identified the dominant environmental factors driving its distribution, and predicted spatiotemporal shifts in its suitable habitats. These results provide a scientific foundation for the conservation of germplasm resources and sustainable management of this species.

## 2 Materials and Methods

### 2.1 Species Distribution Data Preparation and Pre‐Processing

#### 2.1.1 Collection of Occurrence Records for *Sauvagesia rhodoleuca*

**FIGURE 1:**
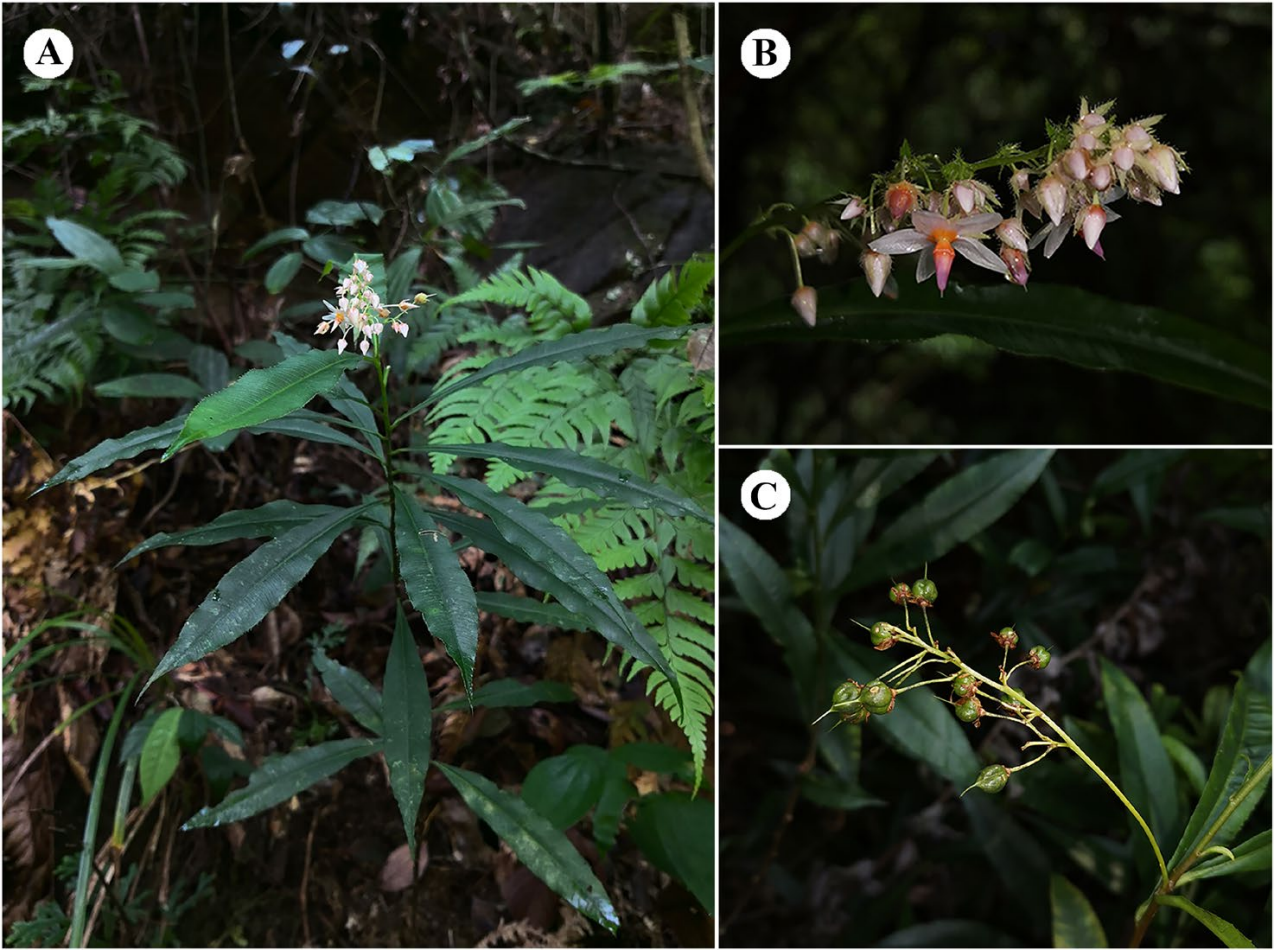
Images of individuals of *Sauvagesia rhodoleuca* in their natural habitats. (A) Plant (B) Flower (C) Fruit.

**FIGURE 2:**
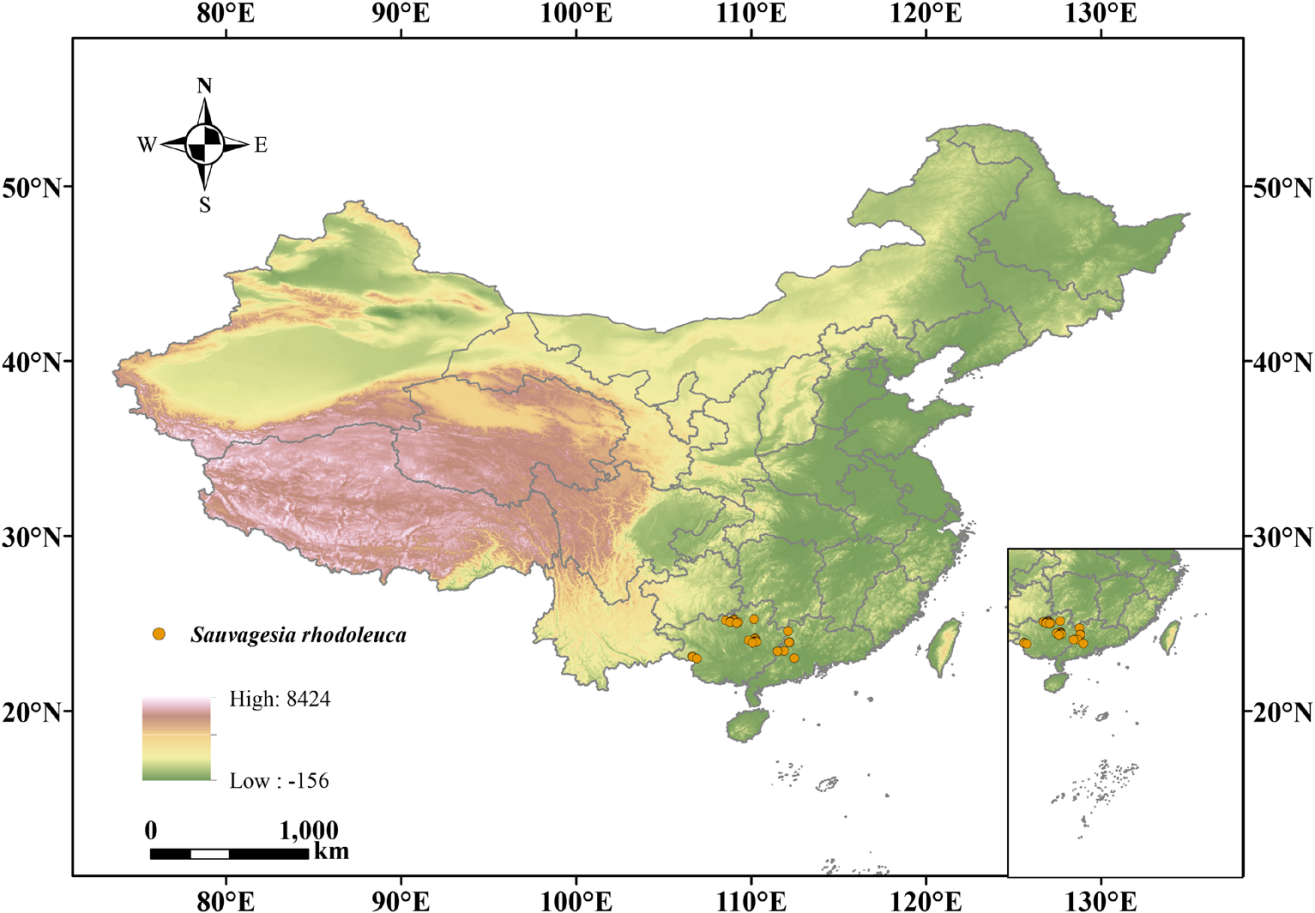
Distribution of *Sauvagesia rhodoleuca* after data cleaning. This map is based on the standard map with review number GS (2024) 0650, and the base map has not been modified.

The geographic distribution data of *S. rhodoleuca* in China were compiled from multiple sources, including the Global Biodiversity Information Facility (GBIF; https://www.gbif.org/, accessed on May 2025), the National Specimen Information Infrastructure (NSII; http://www.nsii.org.cn/, accessed on May 2025), and the Chinese Virtual Herbarium (CVH; http://www.cvh.ac.cn/, accessed on May 2025), supplemented by field surveys. Initially, 31 occurrence records were collected (Figure 1). To reduce spatial sampling bias and minimize clustering effects, we applied spatial filtering using a 30″ resolution grid in ENMTools 1.0 (Warren et al. 2021), resulting in 29 spatially independent occurrence records (saved as CSV files; Figure 2).

#### 2.1.2 Environmental Variable Collection and Screening

In this study, we integrated the influences of climate, topography, soil, and human activities on the distribution of *S. rhodoleuca* and gathered a total of 38 environmental variables (Table 1). Current climate data (1970–2000), future climate projections (for the 2050s–2070s) and elevation data were obtained from the global climate database WorldClim 2.1 (Fick and Hijmans 2017; https://www.worldclim.org/) at a 30″ (1 km × 1 km) resolution. Topographic variables of slope and aspect were derived from the elevation data using ArcGIS software. The future climate data model adopts the three shared socioeconomic path scenarios of SSP126, SSP245 and SSP585 under the medium resolution climate system (BCC‐CSM2‐MR) model of the Sixth International Coupled Model Comparison Program (CMIP6) National (Beijing) Climate Center (Eyring et al. 2016). SSP126 represents a scenario of low greenhouse gas emissions under the sustainable development path. SSP245 represents the scenario of moderate greenhouse gas emissions under the moderate development path. SSP585 represents the scenario of high greenhouse gas emissions under the development path dominated by fossil fuels (Riahi et al. 2017; Meinshausen et al. 2020). Soil variables were obtained from the Harmonized World Soil Database version 2.0 (HWSD v2.0, https://gaez.fao.org/pages/hwsd) at a 30″ resolution. Given that *S. rhodoleuca* is a shallow‐rooted species, only topsoil properties (0–30 cm depth) were selected. Human activities were represented using the human footprint index (Mu et al. 2022). Species distribution projections were made under the assumption that topographic and soil conditions would remain largely unchanged in the future (Zhang et al. 2024). To meet the data format requirements of the MaxEnt model, all environmental variables were processed in ArcGIS to standardize the coordinate system (WGS84), raster cell size, and layer extent. These processed layers were then converted to ASCII format for use in subsequent MaxEnt modeling.

**TABLE 1:** Environmental variables used in the study.

Variables type	Abbreviation	Variable description	Data sources
Bioclimatic variables	Bio1	Annual mean temperature	WorldClim version 2.1 (https://www.worldclim.org/)
	Bio2^a^	Mean diurnal range	
	Bio3	Isothermality	
	Bio4^a^	Temperature seasonality	
	Bio5	Max temperature	
	Bio6	Min temperature of coldest month	
	Bio7	Temperature annual range	
	Bio8^a^	Mean temperature of wettest	
	Bio9	Mean temperature of driest quarter	
	Bio10	Mean temperature of warmest quarter	
	Bio11	Mean temperature of coldest quarter	
	Bio12^a^	Annual precipitation	
	Bio13	Precipitation of wettest month	
	Bio14^a^	Precipitation of driest month	
	Bio15	Precipitation seasonality	
	Bio16	Precipitation of wettest quarter	
	Bio17	Precipitation of driest quarter	
	Bio18^a^	Precipitation of warmest quarter	
	Bio19	Precipitation of coldest quarter	
Topographic variables	Elev	Elevation	WorldClim version 2.1 (https://www.worldclim.org/)
	Slope^a^	Slope	Derived from Elev
	Aspect^a^	Aspect	Derived from Elev
Human variables	HFP^**a**^	Human footprint	Mu et al. (2022)
Soil variables	T_GRAVEL	Topsoil gravel content	Harmonized World Soil Database version 2.0 (https://gaez.fao.org/pages/hwsd)
	T_SAND	Topsoil sand fraction	
	T_SILT	Topsoil silt fraction	
	T_CLAY^a^	Topsoil clay fraction	
	T_REF_BULK_DENSITY	Topsoil reference bulk density	
	T_OC^a^	Topsoil organic carbon	
	T_PH_H2O	Topsoil pH (H2O)	
	T_ESP	Topsoil sodicity (ESP)	
	T_CEC_CLAY	Topsoil CEC (clay)	
	T_BS^a^	Topsoil base saturation	
	T_TEB	Topsoil TEB	
	T_CACO3	Topsoil calcium carbonate	
	T_CASO4	Topsoil gypsum	
	T_ECE	Topsoil salinity (Elco)	
	T_CEC_SOIL	Topsoil CEC (soil)	

^a^ Represents the retained 12 environmental variables.

To mitigate the effects of multicollinearity among the 38 environmental variables with a spatial resolution of 1 × 1 km, we implemented a three‐step screening procedure. First, all environmental variables along with species occurrence data of *S. rhodoleuca* were incorporated into preliminary MaxEnt models (10 replicates). Jackknife tests were employed to evaluate variable contributions, following which factors showing zero contribution were excluded. Subsequently, Pearson correlation analysis was performed using IBM SPSS Statistics 22. Where strong correlations (|*r*| > 0.8) were detected between variable pairs, the variable with lower contribution was removed while prioritizing those with clearer ecological interpretation to minimize multicollinearity (Feng et al. 2019; Xiao et al. 2024). Finally, variance inflation factor (VIF) analysis was conducted on the remaining variables, and only those with VIF values ≤ 5 were retained to further minimize multicollinearity. This screening process resulted in the selection of 12 key environmental predictors for subsequent modeling (Table 1).

### 2.2 Optimization and Evaluation of MaxEnt Models

The complexity of MaxEnt models significantly influences the accuracy of species habitat suitability predictions. Directly using default parameters often leads to overfitting (Warren and Seifert 2011). Therefore, optimizing key parameters is a critical approach to enhancing model performance (Phillips et al. 2006; Morales et al. 2017).

Given the limited distribution points of *S. rhodoleuca* in this study, to minimize the risk of overfitting and enhance model robustness, we did not adopt the default complex parameter combinations designed for large sample sizes. Following common practices for small‐sample modeling, we designed a simplified and strongly constrained parameter optimization process. Specifically, for the regularization multiplier (RM), we tested RM values ranging from 0.5 to 4 in increments of 0.5. For feature combinations (FC), five structural forms including linear (L), quadratic (Q), product (P), threshold (T), hinge (H), and their combinations, were evaluated to control model structural complexity. All parameter combinations were assessed using the ENMeval package in R. Based on the corrected Akaike Information Criterion, the model with the lowest AICc value was selected as the final modeling parameters (Phillips et al. 2017; Zhao et al. 2021).

### 2.3 Model Construction and Evaluation

Based on the optimized regularization multiplier and feature combination parameters, the MaxEnt version 3.4.1 (Phillips et al. 2026) was employed to predict the potential distribution of *S. rhodoleuca* under current conditions and two future time periods. A total of 29 species occurrence records and the 12 selected environmental variables were incorporated into the model. The following parameters were applied: 25% of the occurrence points were randomly selected for testing, with the remaining 75% used for model training. The Bootstrap replication method was selected with 10 replicates. The distribution values were output in logistic form, and the average prediction across the 10 replicates was used as the final result to reduce model stochasticity and simulation uncertainty (Wan et al. 2021; Moreno et al. 2011). The Jackknife test was applied to evaluate the contribution of each environmental variable. Dominant factors were identified based on three metrics: percent contribution, permutation importance, and Jackknife test results.

To calibrate the model and assess its robustness, the predictive performance of the MaxEnt model was evaluated using the area under the curve (AUC) and the true skill statistic (TSS). The AUC value ranges from 0.5 to 1 and serves as a key indicator of prediction performance: 0.5–0.6 indicates a failed model, 0.6–0.7 poor, 0.7–0.8 fair, 0.8–0.9 good, and 0.9–1 excellent. Generally, AUC values closer to 1 signify better predictive performance (Ouyang et al. 2019; Li et al. 2018). The TSS ranges from −1 to 1, with values closer to 1 indicating superior predictions. A TSS between 0.6 and 1 is considered to represent good predictive capability (Allouche and Kadmon 2006).

### 2.4 Classification and Statistical Analysis of Suitable Habitats

The potential habitat suitability of *S. rhodoleuca* was rationally categorized using a multi‐threshold approach (Luo et al. 2024; Liu et al. 2025). First, the balance training omission, predicted area, and threshold value (BTPT) was employed to delineate suitable and unsuitable habitats. Subsequently, the maximum training sensitivity plus specificity (MTSS) threshold was selected to define moderately suitable habitats. To identify highly suitable habitats, the Jenks natural breaks method was applied to the continuous habitat suitability output, generating four classes, with the third breakpoint serving as the lower threshold and 1 as the upper limit. Using the reclassification tool in ArcGIS, the potential habitat suitability of *S. rhodoleuca* was classified into four categories: nonsuitable area (*p* ≤ 0.0346), low‐suitability area (0.0346 < *p* ≤ 0.3523), medium‐suitability area (0.3523 < *p* ≤ 0.5195), and high‐suitability area (0.5195 < *p* ≤ 1). To ensure the comparability of habitat suitability across different spatiotemporal scales, the identical thresholds were used for both the current and future periods. To analyze future changes in suitable areas, SDMToolbox was used to classify habitat suitability changes for *S. rhodoleuca* into four types: no, gain, stable, and loss. Then calculate the number of grids in each class, and then convert the area of each class based on the area of the cropped grids (Jiang et al. 2023).

### 2.5 Changes in Potential Risk Areas and Centroid Shifts

The SDMtools toolbox in ArcGIS was employed to analyze shifts in suitable habitat distribution for *S. rhodoleuca*. Continuous raster data from different periods were converted into binary format using the “Quick Reclassify to Binary” tool. The “Centroid Changes” tool was then applied to trace the trajectory of centroid movement in suitable habitats. Finally, the “Distribution Changes Between Binary SDMs” tool was used to compare the areal extent of suitable habitats between current and future scenarios, and to evaluate patterns of expansion and contraction in the species' national distribution under future climate conditions, thereby elucidating overall distribution trends.

## 3 Results

### 3.1 Model Optimization and Accuracy Assessment

Based on systematic parameter optimization and model evaluation, the parameter configuration with RM = 3.5 and FC = LQHP was identified as the optimal model for predicting the potential distribution of *S. rhodoleuca*. This model exhibited the lowest delta AICc value (delta AICc = 0) among all tested combinations and was therefore selected for subsequent analysis. The optimized model demonstrated high performance and stability across 10 replicate runs, with AUC = 0.988 and TSS = 0.73 for the training dataset. These results indicate that the model achieves a balance between controlled complexity and robust predictive accuracy, along with strong generalization capability.

### 3.2 Environmental Variables Influencing the Spatial Distribution of *Sauvagesia rhodoleuca*

**FIGURE 3:**
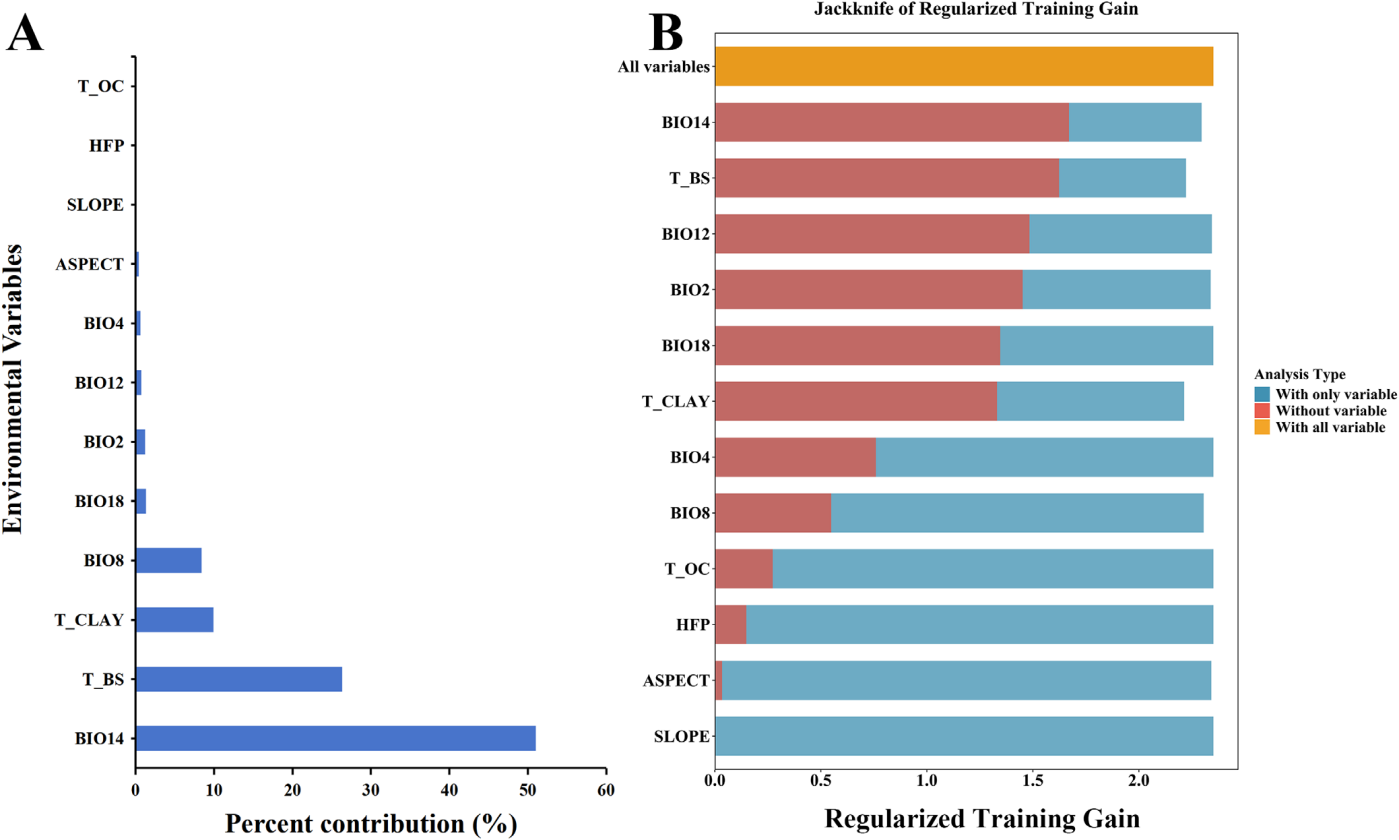
(A) Contribution, (B) Jackknife evaluation of regularized training gain of environmental variables associated with *Sauvagesia rhodoleuca* via MaxEnt.

Based on the results of the jackknife test and percent contribution, this study comprehensively evaluated the influence of climatic variables on the suitable habitat distribution of *S. rhodoleuca* (Xiang et al. 2025). The relationships between the probability of *S. rhodoleuca* occurrence and the dominant climatic factors were further analyzed using response curves. The top 5 environmental variables ranked by percent contribution are as follows (Figure 3A): Bio14 (51.0%), T_BS (26.3%), T_CLAY (9.9%), Bio8 (8.4%), Bio18 (1.3%). The 12 environmental variables can be categorized into four groups: climate, soil, topography, and human activity. Among them, climate and soil factors dominated the model explanation, with cumulative contribution rates of 63.2% and 36.2%, respectively. Topography (0.5%) and human activity (0%) have a negligible impact on it. The regularized training gain from the jackknife test (Figure 3B) indicated that when used individually, the six variables with the highest gains were, in descending order: Bio14, T_BS, Bio12, Bio2, Bio18, and T_CLAY. Based on the criterion of cumulative contribution rate exceeding 90.4% (Yang et al. 2022), Bio14, T_BS, T_CLAY, and Bio8 were identified as the four key environmental variables, with a cumulative contribution rate of 95.6%, playing a significant regulatory role in the potential distribution pattern of *S. rhodoleuca* in China.

**FIGURE 4:**
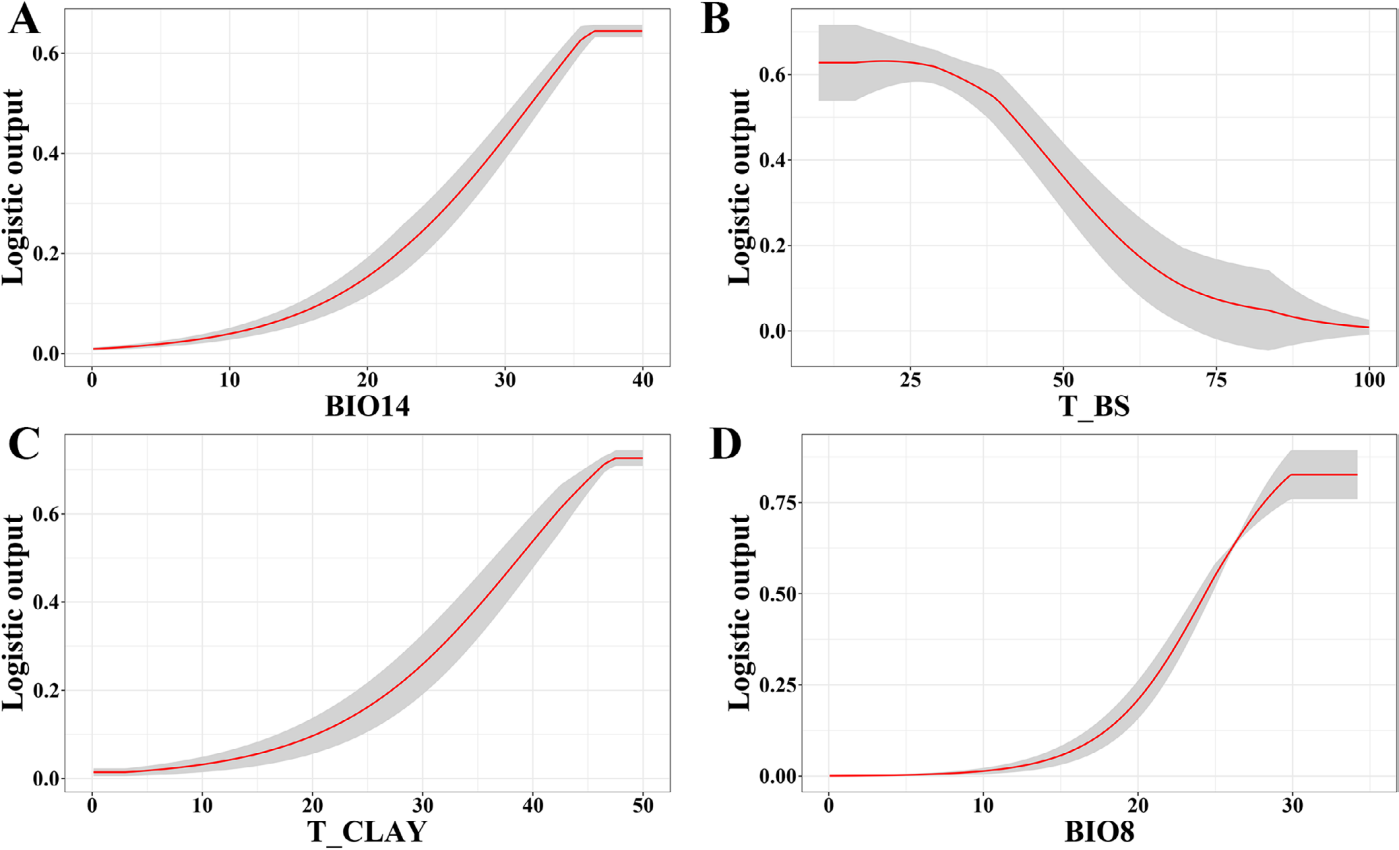
Response curves of the key environmental variables.

Response curves of individual environmental variables further elucidated the correlative trends between the occurrence probability of *S. rhodoleuca* and the dominant factors. An occurrence probability greater than 0.5 indicates that the corresponding environmental conditions are favorable for the species (Gan et al. 2024). Based on the response curves of the dominant factors (Figure 4), precipitation of the driest month (Bio14), topsoil base saturation (T_BS), percentage clay in topsoil (T_CLAY), and mean temperature of the wettest quarter (Bio8) all exhibited monotonic relationships with the distribution probability of *S. rhodoleuca*: Bio14, T_CLAY, and Bio8 showed monotonically increasing trends, while T_BS exhibited a monotonically decreasing trend. The probabilities peaked at values of 36.5 mm, 20.1%, 47.6% wt. and 30.0°C, respectively. When the precipitation of the driest month is below 32 mm, the species occurrence probability rises significantly with increasing precipitation. When precipitation exceeds 32 mm, the probability stabilizes at a level between 0.6 and 0.7. When T_BS is higher than 41.7%, the species occurrence probability shows a decreasing trend from 0.5; this suggests that heavily salinized soils impose substantial stress on the species' survival, highlighting the species' stringent demands for soil fertility. When T_CLAY rises from 38.8% to 47.6% wt., and Bio8 rises from 24.4°C to 30°C, the species occurrence probability rises from 0.5 and then enters a stable plateau.

### 3.3 Suitable Distribution Areas of *Sauvagesia rhodoleuca* in China Under Current and Future Climate Scenarios

**FIGURE 5:**
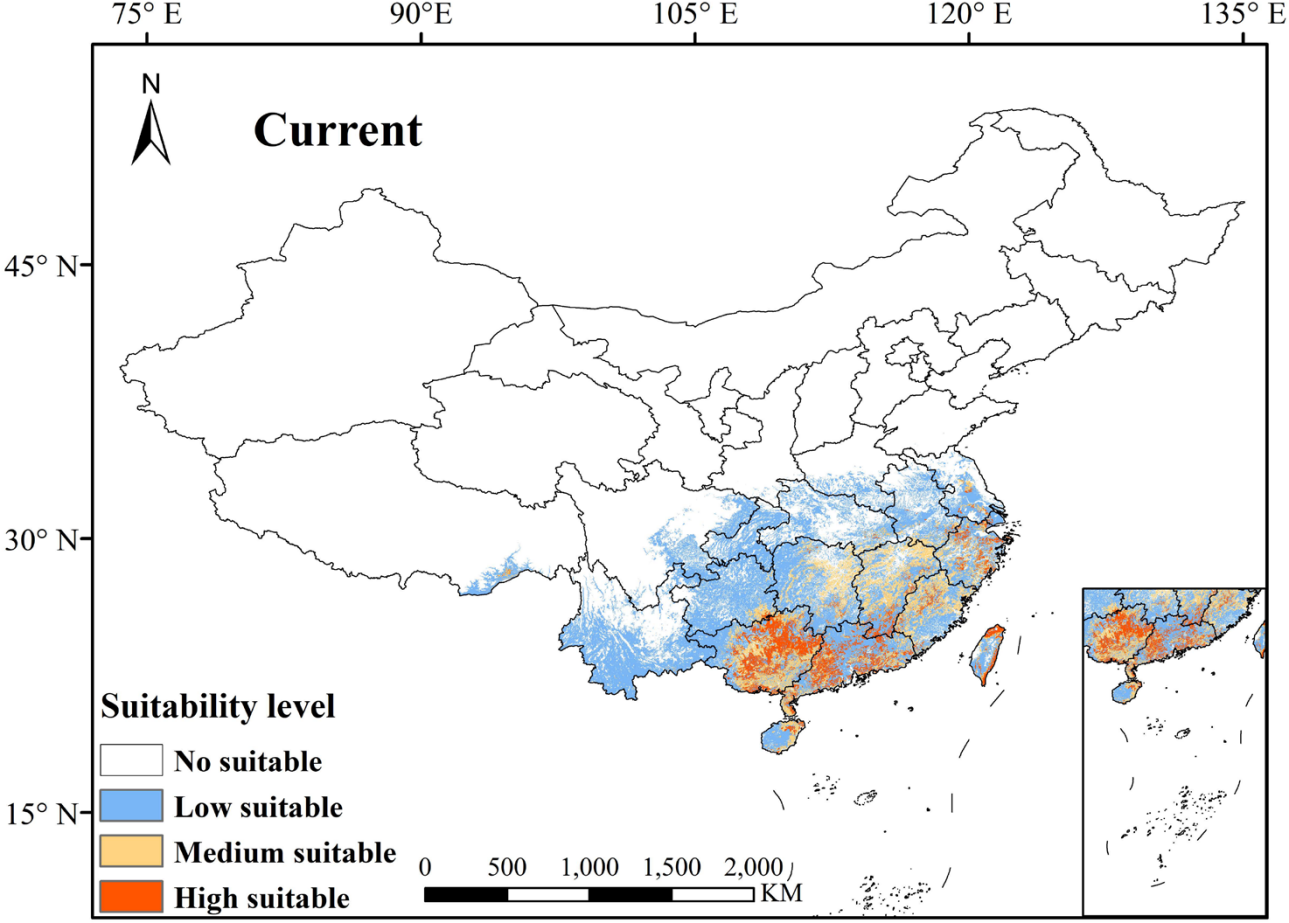
Potential geographical distribution of *Sauvagesia rhodoleuca* in the current climatic environment.

Under current climatic conditions, the total suitable habitat area for *S. rhodoleuca* is approximately 146.81 × 10^4^ km^2^ (Figure 5 and Table 2), accounting for about 15.29% of China's total land area. Highly suitable areas are predominantly concentrated in most regions of Guangxi and Guangdong, with fragmented patches distributed in Fujian, Jiangxi, Hainan, Taiwan, Zhejiang, Jiangsu, Anhui, Hubei, Hunan, Chongqing, Sichuan, Guizhou, Yunnan, and Xizang. Among these, the area of highly suitable habitat covers about 14.29 × 10^4^ km^2^, moderately suitable habitat comprises approximately 28.29 × 10^4^ km^2^, and lowly suitable habitat spans about 104.23 × 10^4^ km^2^, representing 1.49%, 2.94%, and 10.86% of China's terrestrial area, respectively. A comparison between the predicted results and the actual occurrence records of *S. rhodoleuca* shows that all known distribution points fall within the predicted suitable areas, further supporting the reliability of the model projections.

**TABLE 2:** Area of suitable habitats for *Sauvagesia rhodoleuca* under current and future climate scenarios.

Chronology and climate scenarios	Low **suitability** area (10^4^ km^2^)	Medium suitability area (10^4^ km^2^)	High suitability area (10^4^ km^2^)	Total suitable area (10^4^ km^2^)
Current	104.23	28.29	14.29	146.81
2050s‐SSP126	124.24	31.68	45.87	201.79
2070s‐SSP126	113.84	28.82	41.75	184.41
2050s‐SSP245	121.94	35.25	42.84	200.03
2070s‐SSP245	119.86	34.79	43.20	197.84
2050s‐SSP585	109.68	31.25	62.69	203.62
2070s‐SSP585	126.27	37.04	77.17	240.48

**FIGURE 6:**
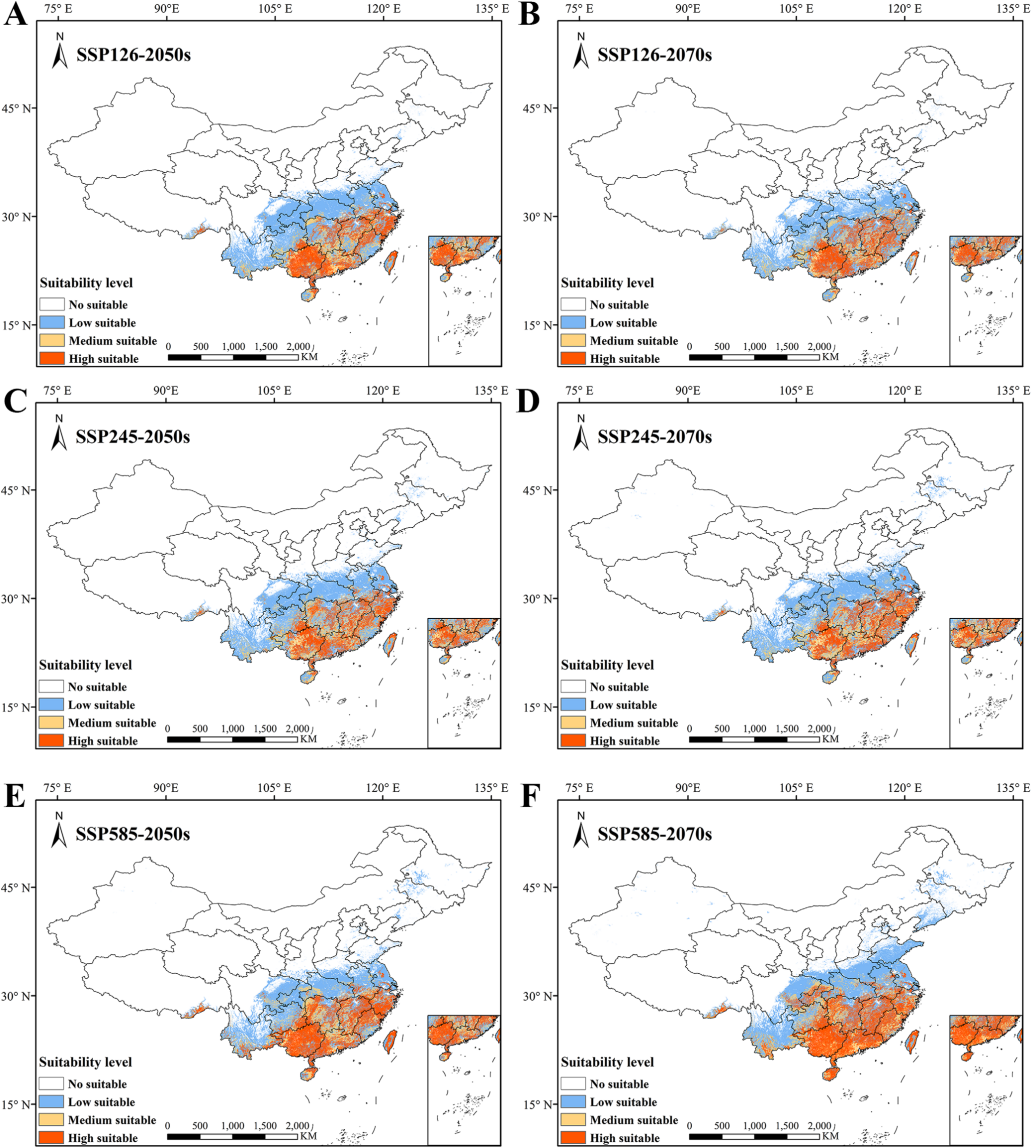
Potential geographical distribution of *Sauvagesia rhodoleuca* in the future climatic environment. (A) SSP126 in the 2050s; (B) SSP126 in the 2070s; (C) SSP245 in the 2050s; (D) SSP245 in the 2070s; (E) SSP585 in the 2050s; (F) SSP585 in the 2070s.

Projections of the potential distribution of *S. rhodoleuca* across China under three representative emission scenarios (SSP126, SSP245, and SSP585) for the periods 2050s and 2070s indicate varied expansions in suitable habitat compared to current climatic conditions (Figure 6 and Table 2). By the 2050s, the total suitable habitat area is projected to increase to 201.79 × 10^4^ km^2^ under the SSP126 scenario, 200.03 × 10^4^ km^2^ under the SSP245 scenario, and 203.62 × 10^4^ km^2^ under the SSP585 scenario, representing expansions of 37.45%, 36.25%, and 38.70% relative to the current total suitable area, respectively. By the 2070s, the total suitable habitat area is projected to increase to 184.41 × 10^4^ km^2^ under the SSP126 scenario, 197.84 × 10^4^ km^2^ under the SSP245 scenario, and 240.48 × 10^4^ km^2^ under the SSP585 scenario, representing expansions of 25.62%, 34.76%, and 63.81% relative to the current total suitable area, respectively. Compared with the current period, the most substantial increases in specific suitability classes are all observed under SSP585 in the 2070s: the lowly suitable area expands by 22.05 × 10^4^ km^2^, the medium suitable area expands by 8.75 × 10^4^ km^2^; the highly suitable area increases most under SSP585, rising by 62.87 × 10^4^ km^2^.

### 3.4 Changes in the Spatial Pattern of Future Suitable Distribution Areas

**FIGURE 7:**
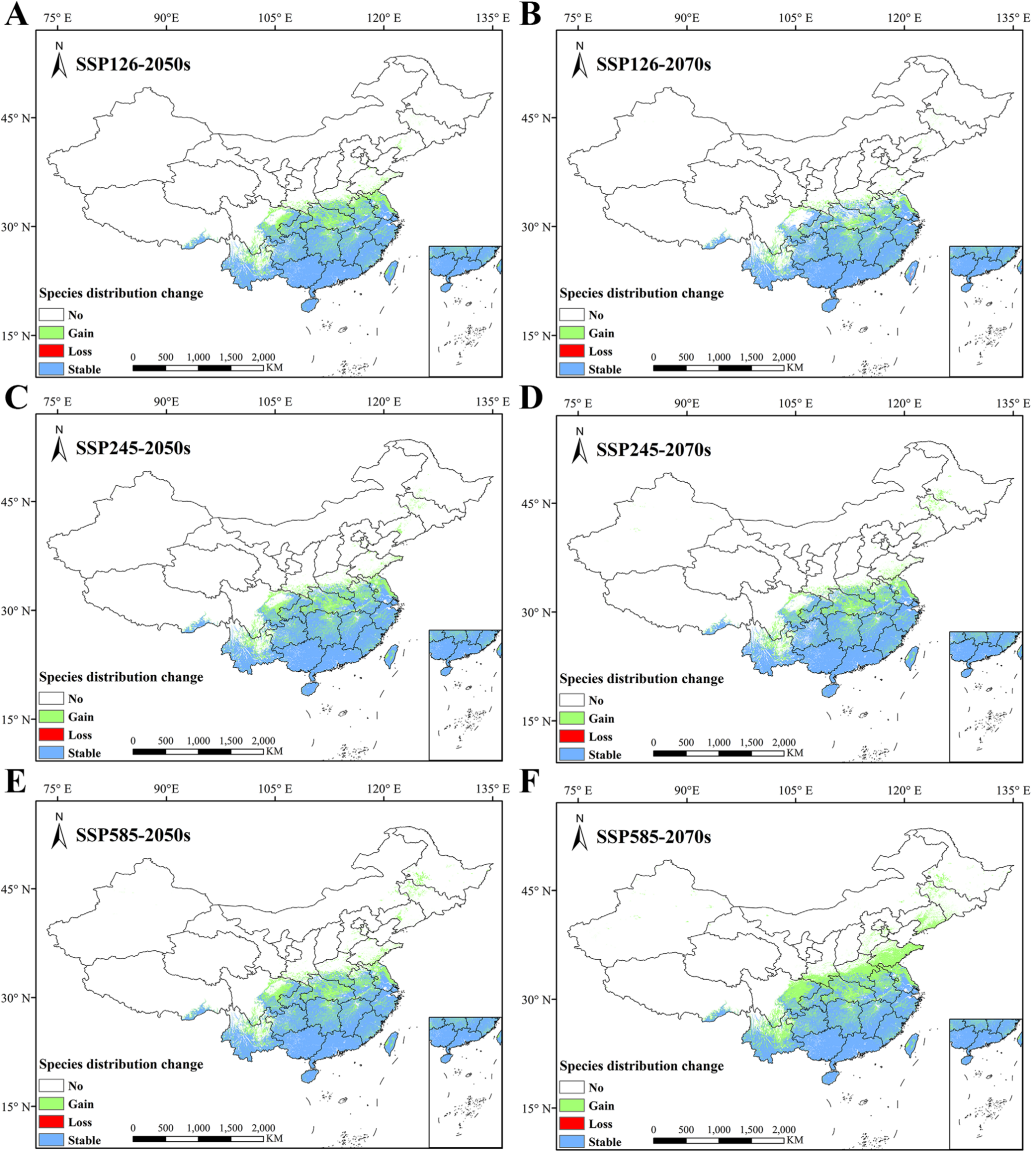
Spatial pattern changes of *Sauvagesia rhodoleuca* under future climate change scenarios. (A) SSP126 in the 2050s; (B) SSP126 in the 2070s; (C) SSP245 in the 2050s; (D) SSP245 in the 2070s; (E) SSP585 in the 2050s; (F) SSP585 in the 2070s.

The spatial patterns of suitable habitat distribution for *S. rhodoleuca* under two future climate scenarios (2050s and 2070s) were compared and analyzed (Figure 7). The results indicate that under future climate change, the suitable habitat areas of *S. rhodoleuca* expand to varying degrees across all scenarios, with the magnitude of expansion substantially exceeding that of contraction (Table 3). Under the three future climate scenarios, *S. rhodoleuca* retains the majority of its current suitable habitat. The only habitat loss occurs under the SSP126 scenario in the 2070s, amounting to 0.09 × 10^4^ km^2^, representing a 0.06% loss. All periods and scenarios exhibit habitat gains and losses to different extents. The highest habitat gain occurs under the SSP585 scenario in the 2070s, expansion by 93.67 × 10^4^ km^2^, which corresponds to a 63.81% increase. The smallest gain is observed under the SSP126 scenario in the 2070s, with an expansion of 37.7 × 10^4^ km^2^, equivalent to a 25.68% increase. Overall, the future potential distribution of *S. rhodoleuca* demonstrates a trend of “northward expansion,” indicating that climate change is likely to drive a shift of its suitable habitats toward northern regions.

**TABLE 3:** Changes in the distribution area of *Sauvagesia rhodoleuca* across different periods under various climate scenarios.

Chronology and climate scenarios	Area/(×10^4^ km^2^)			Area change rate/%		
	Gain	Stable	Loss	Increasing rate	Attrition rate	Retention rate
2050s‐SSP126	54.99	146.81	0	37.45	0	100
2070s‐SSP126	37.7	146.72	0.09	25.68	0.06	99.94
2050s‐SSP245	53.22	146.81	0	36.25	0	100
2070s‐SSP245	51.03	146.81	0	34.76	0	100
2050s‐SSP585	56.82	146.81	0	38.7	0	100
2070s‐SSP585	93.67	146.81	0	63.81	0	100

### 3.5 Shift in the Centroid of Suitable Habitats for *Sauvagesia rhodoleuca* in China Under Climate Change Scenarios

**FIGURE 8:**
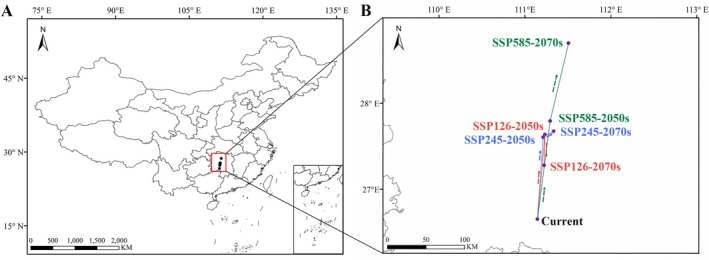
Geographical distribution changes in the centroid of the suitable growing area of *Sauvagesia rhodoleuca* under different climate change scenarios. (A) Overall migration trajectories of centroids under current and future climate scenarios (2050s and 2070s). (B) Enlarged view of centroid migration trajectories.

The centroid of suitable habitats for *S. rhodoleuc*a exhibits directional variation but shows an overall northward shift across different periods and climate scenarios (Figure 8). Under current conditions, the centroid is located at 111.14° E, 26.65° N. Under the SSP126 scenario, the centroid is positioned at 111.23° E, 27.64° N in the 2050s and shifts southeastward to 111.22° E, 27.27° N by the 2070s. Under the SSP245 scenario, the centroid moved northeast from 111.21° E, 27.61° N in the 2050s to 111.33° E, 27.68° N in the 2070s. Similarly, under the SSP585 scenario, the centroid is located at 111.29° E, 27.79° N in the 2050s and shifts northeast to 111.50° E, 28.69° N in the 2070s. Compared to the current centroid, the greatest northeastward displacement occurs under the SSP585 scenario in the 2070s, and the northernmost position overall is observed under the SSP585 scenario in the 2070s as well. Although southeastward shifts occur within the SSP126 scenarios in the 2070s, the centroid under all future scenarios is located north of its current position. This pattern suggests that future climate warming and increased humidity may drive a northward shift in the centroid of suitable growing regions for *S. rhodoleuca*.

## 4 Discussion

### 4.1 Parameter Optimization of the MaxEnt Model and Reliability of Prediction Results

The reliability of ecological niche model predictions depends primarily on three aspects: model selection, sample size and coverage, and the type and number of environmental factors (Anderson and Gonzalez 2011; Elith et al. 2011; Merow et al. 2013). Regarding model selection, different ecological niche models exhibit significant variations in predictive accuracy. Among them, the MaxEnt model is widely used for species with narrow distribution ranges, such as endangered species and rare medicinal plants, due to its low sample size requirements, high predictive accuracy, and strong stability (Phillips et al. 2006; Elith et al. 2011). It is noteworthy that optimizing the regularization multiplier (RM) and feature class (FC) parameters of the MaxEnt model using the R package ENMeval can significantly improve model performance (Muscarella et al. 2014; Kass et al. 2021). Compared with the default parameters, the optimized model reduces the risk of overfitting, enhances the reliability of predictions, and produces smoother response curves that more accurately reflect the species' response to environmental variables (Warren and Seifert 2011; Radosavljevic and Anderson 2014; Zhu and Qiao 2016). In this study, we used the ENMeval package to optimize the MaxEnt model parameters and ultimately determined the optimal configuration: RM = 3.5 and FC = LQHP. This configuration achieved exceptionally high predictive accuracy (AUC ≥ 0.988; TSS = 0.73) under both current and future climates, and all actual distribution points of *S. rhodoleuca* under current climatic conditions fell within the predicted suitable areas, further validating the reliability of the model predictions.

In terms of sample data, a sufficient sample size can minimize the impact of sampling bias on model performance, and the closer the sample distribution is to the species' actual distribution, the higher the predictive accuracy (Hernandez et al. 2006; Wisz et al. 2008). In this study, we scientifically screened the distribution data of *S. rhodoleuca* to ensure that the collected samples covered the species' known distribution range, thereby meeting the data requirements for spatial prediction in the MaxEnt (Pearson et al. 2007; Kramer‐Schadt et al. 2013).

Regarding environmental factors, the rationality of the selected environmental variables in the MaxEnt model directly affects the simulation outcomes. The more comprehensive the factors related to the ecological niche, the more reliable the simulation results (Guisan and Thuiller 2005). Based on the ecological characteristics of *S. rhodoleuca*, this study systematically selected key environmental variables to lay a solid foundation for reliable model simulations.

### 4.2 Influence of Dominant Environmental Factors on the Distribution of *Sauvagesia rhodoleuca*

MaxEnt model results indicate that the distribution of *S. rhodoleuca* is primarily determined by precipitation (Bio12, Bio14, Bio18), temperature (Bio2, Bio4, Bio8), soil factors (T_BS, T_CLAY), topography (Aspect, Slope), and human activity (HFP). The cumulative contribution of climatic factors reached 63.2%, substantially higher than that of the soil factor 36.2%, indicating that hydrothermal conditions are the primary constraints on the species distribution, soil factors play a secondary regulatory role. This finding aligns with general biogeographical principles, which emphasize that temperature and precipitation shape species distribution patterns by influencing key physiological processes such as photosynthesis, water‐use efficiency, and seed germination (Li et al. 2013; Zhu et al. 2013).

*S. rhodoleuca* grows within the latitude range of 23° N to 25.3° N in China, and its physiological characteristics exhibit a strong dependence on highly humid environments (Chai et al. 2010). Bio14 emerged as the most influential variable, with an optimal range higher than 32.0 mm, indicating that this species has strong adaptability to humid environments, while drought conditions are the main limiting factor for its distribution (Chen et al. 2016). This may be linked to its unique phenology: seeds are dispersed in September–October, a season characterized by declining temperature and rainfall, and remain dormant until late spring or early summer when conditions become favorable for germination (Chai et al. 2010). Insufficient dry‐season rainfall may directly impede seedling recruitment, leading to population decline. Bio8 contributed 8.4%, with an optimum range higher than 24.4°C, closely matching the thermal window for seed germination. Empirical studies report optimal germination at 25°C and strong suppression below 20°C (Chai et al. 2010).

The suitable range of T_BS is 7.6%–41.7% and shows a positive correlation with germination rate. Studies indicate that germination is optimal at 20%–30% soil moisture, with higher moisture levels accelerating germination (Chai et al. 2010). T_CLAY contributed 9.9%, indicating a significant role in fine‐scale habitat suitability. Clay soils have better water and nutrient retention capacity, which can minimize water loss and nutrient leaching, critical for germination and early seedling survival (Miao et al. 2020). The small seed size and limited nutrient reserves of *S. rhodoleuca* make seedlings highly dependent on external nutrient availability, which may be more accessible in clay soils due to better root‐soil contact and microbial activity (Chai et al. 2010).

The topographical factors have almost no effect on the distribution of *S. rhodoleuca*, which may be because its primary growth region (23° N–25.3° N) is mostly characterized by low mountains, hills, and plains with little topographical variation. The spatial heterogeneity of slope and aspect is insufficient to create significant habitat filtering. Additionally, this area belongs to a subtropical monsoon climate, where water and heat resources are abundant and relatively evenly distributed. Climate factors (especially precipitation) directly meet the species' needs, and the role of topography in the redistribution of water and heat resources is not significant.

In summary, climatic factors (temperature and precipitation) dominate the distribution of *S. rhodoleuca* at broad scales, explaining 63.2% of the model variance, while soil properties also demonstrate a notable impact (36.2%). Topography and human activity showed minimal contributions of only 0.5% and 0% respectively, suggesting their limited direct influence on the species' large‐scale distribution patterns. This hierarchical control mechanism highlights that while macroclimatic conditions set the fundamental range boundaries, soil properties fine‐tune habitat suitability at local scales, whereas topographic and anthropogenic factors play secondary roles in shaping the species' distribution. Research has proven that under future climate scenarios, models including soil predicted much smaller northward shifts in distributions than climate‐only models (Ni and Vellend 2023). An integrated perspective that considers multi‐scale interactions among climate, soil, and topography is essential for accurately predicting species' range dynamics under environmental change.

### 4.3 Changes in *Sauvagesia rhodoleuca* Potential Distribution Under Climate Change Scenarios and Their Driving Factors

This study demonstrates that the potential suitable area of *S. rhodoleuca* shows an increasing trend under different future climate scenarios. This finding is consistent with recent studies reporting climate change‐induced range expansion of the species (Wu et al. 2022; He et al. 2023). According to IPCC data, the global mean temperature increased by approximately 0.74°C between 1906 and 2005, and China's regional mean temperature rose by about 1.1°C from 1951 to 2001, with the northern boundary of the subtropics shifting northward by up to 120 km. Meanwhile, China's regional mean annual precipitation is projected to increase by 0%–20% in the future (Jiang and Fu 2012), with the climate generally trending toward warming and wetting. As a plant group inhabiting the northern edge of the southern subtropics and the southern edge of the central subtropics, *S. rhodoleuca* benefits from global warming, which facilitates the expansion of its potential distribution range in China. Current and future distribution predictions show that *S. rhodoleuca* is endemic to the subtropical monsoon climate zone, and its distribution northern boundary aligns with the zone's northern limit, confirming that the subtropical monsoon climate's mild and humid conditions meet its temperature and moisture requirements. Bio14 and Bio8 are key climate factors governing *S. rhodoleuca* distribution, highlighting their role in future habitat expansion.

With global warming, the boundary of the subtropical monsoon climate is shifting northward, accompanied by changes in precipitation and temperature within the current distribution area. Studies have shown that the distribution center of *S. rhodoleuca* tends to migrate northward in the future, indicating its adaptation to climate change by spreading to higher latitudes (Parmesan and Yohe 2003; Root et al. 2003; Lenoir et al. 2010). Many species have expanded their potential habitats toward higher latitudes, which is a common adaptation strategy for plants to cope with climate change by adjusting their distribution (Chen et al. 2011). The warming and humidifying climate provides a wider range of suitable areas for *S. rhodoleuca* distributed in the northern edge of the southern subtropics and the southern edge of the central subtropics.

### 4.4 Strategies for Germplasm Conservation of *Sauvagesia rhodoleuca*

Although model predictions indicate that climate change may expand the potential habitat of *S. rhodoleuca*, the species' actual survival prospects remain grim. As a rare plant with significant medicinal value, its rhizomes have long been overexploited, and combined with deforestation and habitat destruction, has led to the decline of wild populations, putting them at risk of extinction (Miao et al. 2020). This paradox of “predicted habitat expansion versus actual population decline” underscores the urgency of enhancing conservation efforts for this species.

This study identifies the Dayao Mountain and Jiuwan Mountain Nature Reserves in Guangxi as stable high‐suitability habitats for *S. rhodoleuca*, making them priority sites for carrying out the reconstruction of wild germplasm resources and designated as key areas for introducing planting cultivation. In addition, with climate warming promoting the northward expansion of suitable areas, newly suitable regions like Hunan and Jiangxi can be targeted for introducing *S. rhodoleuca* to expand its planting range. It is worth noting that, according to the “adversity theory” (Huang and Guo 2007), the optimal environment for plant growth may not coincide with, or may even be contrary to, the best conditions for the accumulation of medicinal compounds. From the perspective of medicinal material utilization, when planning the production areas of *S. rhodoleuca*, it is necessary not only to use habitat suitability to identify environments favorable for its growth, but also to consider whether the region is suitable for the accumulation of its active compounds (Liu et al. 2016; Jiang et al. 2023).

### 4.5 Limitations and Future Perspectives

The MaxEnt model used in this study primarily predicts the fundamental ecological niche requirements of species based on abiotic factors, while the actual ecological niche is also influenced by biotic factors (e.g., interspecific relationships). Since no single model can account for all influencing factors, the simulation results only represent the potential distribution range under ideal conditions (Qiao et al. 2013; Chakraborty et al. 2016).

The limited sample size employed in this study may constrain the robustness of model predictions. Sufficient sample coverage is critical to minimizing sampling bias and enhancing the accuracy of species distribution modeling, and the current sample scale may not fully eliminate such potential biases (Hernandez et al. 2006; Wisz et al. 2008).

This study focused solely on analyzing the ecological suitability for *S. rhodoleuca* growth and classifying habitat suitability, but the optimal growth environment does not guarantee the effective accumulation of medicinal components. Thus, the current findings cannot fully address the comprehensive needs for the conservation and sustainable utilization of this species.

The low contribution of human activity (HFP) should be interpreted cautiously, as our model used data is a summary of 8 variables that can reflect different aspects of human pressures. This may weaken the influence of a specific human activity on the distribution of *S. rhodoleuca*. Future studies with fine‐scale human disturbance metrics may reveal more significant impacts on local populations.

To improve prediction accuracy, future studies could adopt a multi‐model integration strategy—for example, combining generalized linear models (GLM) with gradient boosting models (GBM)—and incorporate biotic factors (e.g., intraspecific/interspecific interactions). Integrating multidimensional ecological process information will significantly enhance the reliability of species distribution predictions, providing a more robust theoretical basis for formulating conservation strategies for endangered species (Feeley and Silman 2010; Zhou et al. 2023).

Future research should further explore the quantitative relationship between environmental factors and the accumulation of medicinal active ingredients in *S. rhodoleuca*. Combining habitat suitability analysis with quality suitability assessment will provide more comprehensive scientific support for the conservation and sustainable utilization of this species.

## 5 Conclusion

This study utilized the MaxEnt model to comprehensively analyze the climatic, topographic, and soil factors influencing the natural distribution of *S. rhodoleuca*. The results showed that the AUC values of the MaxEnt model were all above 0.988, indicating high predictive accuracy and reliability. Further analysis identified Bio14, T_BS, T_CLAY, and Bio8 as the dominant factors shaping the potential distribution of *S. rhodoleuca*, with hydrothermal conditions playing a primary role in determining its geographic range. Current climate simulations indicate that the species is primarily distributed in regions such as Guangxi and Guangdong, which is consistent with its known actual distribution. In addition, simulations under future climate change scenarios suggest that, under trends of rising temperature and increased precipitation, the suitable habitats of *S. rhodoleuca* may significantly expand and shift toward higher latitudes. By modeling the potential distribution and ecological suitability of *S. rhodoleuca* under different climate conditions, this study not only provides a theoretical basis for the conservation of its wild germplasm resources but also offers valuable references for its cultivation, introduction, and rational regional planning.

## Author Contributions

**Jinxin Wei:** conceptualization (equal), data curation (equal), investigation (equal), methodology (equal), software (equal), writing – original draft (equal), writing – review and editing (equal). **Bangze Li:** data curation (equal), formal analysis (equal), software (equal), writing – original draft (equal). **Rong Zou:** methodology (equal), supervision (equal). **Weiping Wu:** investigation (equal), validation (equal). **Fei Tan:** investigation (equal), supervision (equal). **Li Ding:** supervision (lead), writing – review and editing (equal). **Tao Ding:** funding acquisition (lead), methodology (equal), writing – review and editing (equal).

## Funding

This study was supported by the Guangxi Forestry Science and Technology Promotion Demonstration project (No. 2022GT23), Guangxi Natural Science Foundation Project (No. 2025GXNSFAA069161), and Guangxi Institute of Botany Discipline Development Fund Project (No. GZF030).

## Conflicts of Interest

The authors declare no conflicts of interest.

## Data Availability

The dataset used in this study is publicly available from the Dryad Digital Repository (https://datadryad.org/dataset/doi:10.5061/dryad.dz08kpsbf).
